# Pyrolyzed Parylene-N for *in Vivo* Electrochemical Detection of Neurotransmitters

**DOI:** 10.1021/acselectrochem.4c00180

**Published:** 2025-03-27

**Authors:** He Zhao, Owen Markow, Greatness Olaitan, Eric D. Donarski, Kevin C. Lester, Nickolay V. Lavrik, B. Jill Venton

**Affiliations:** † Department of Chemistry, University of Virginia, Charlottesville, Virginia 22904, United States; ‡ Center for Nanophase Materials Sciences, Oak Ridge National Lab, Oak Ridge, Tennessee 37831, United States

**Keywords:** Parylene-N, Carbon electrode, Adsorption, Antifouling, *In vivo*

## Abstract

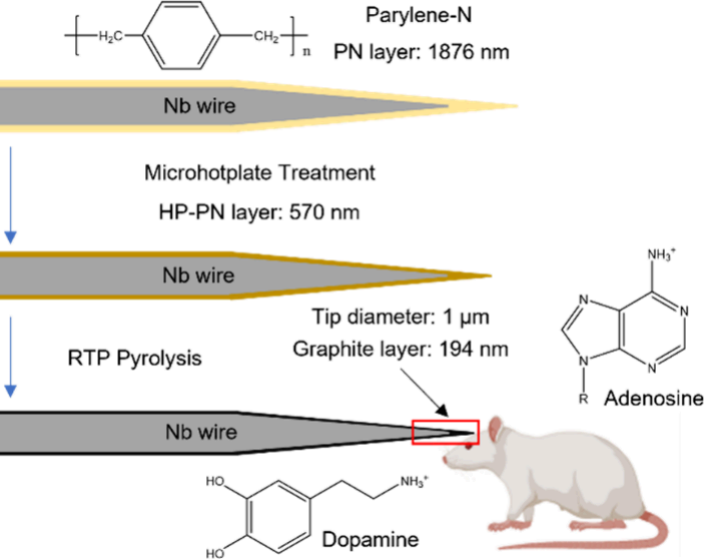

Carbon electrodes are typically used for *in vivo* dopamine detection, and new types of electrodes and customized fabrication methods will facilitate new applications. Parylene is an insulator that can be deposited in a thin layer on a substrate and then pyrolyzed to carbon to enable its use as an electrode. However, pyrolyzed parylene has not been used for the real-time detection of neurochemicals by fast-scan cyclic voltammetry. In this work, we deposited thin layers of parylene-N (PN) on metal wires and then pyrolyzed them to carbon with high temperatures in a rapid thermal processor (RTP). Different masses of PN, 1, 6, and 12 g, were deposited to vary the thickness. RTP-PN (6 g) produced a 194 nm layer carbon thickness and had optimal electrochemical stability. Pyrolyzed parylene-N modified electrodes (PPNMEs) were characterized for electrochemical detection of dopamine, serotonin, and adenosine. Background-normalized currents at PPNMEs were about 2 times larger than those of carbon-fiber microelectrodes (CFMEs). Rich defect sites and oxygen functional groups promoted the neurochemical adsorption of cationic neurotransmitters. PPNMEs resisted fouling from serotonin polymer formation. PPNMEs were used *in vivo* to detect stimulated dopamine release and monitor spontaneous adenosine release. Pyrolyzed parylene is a sensitive and fouling-resistant thin-film carbon electrode that could be used in the future for making customized electrodes and devices.

## Introduction

Carbon-fiber microelectrodes (CFMEs) are commonly used for the electrochemical detection of monoamine neurotransmitters with fast-scan cyclic voltammetry (FSCV). CFMEs are applied for *in vivo* or *in vitro* tracking of neurochemicals because of the biocompatibility and relatively small diameter (∼7 μm). However, different types of carbon electrodes have been developed to enhance the electrochemical properties for many applications. For example, carbon nanotubes (CNTs) in CNT yarn microelectrodes efficiently trap neurochemicals because of thin-layer effects.^1−3^ Carbon nanospikes (CNSs) are deposited on niobium (Nb) wires and utilized to promote analyte adsorption and resist fouling.^4−6^ Pyrolyzed photoresist electrodes were developed to take advantage of thin polymer films deposited on wires or other substrates to make arrays.^7^ Tour’s group demonstrated laser pyrolysis of polymers to make electrode materials.^8,9^ Many pyrolyzed carbon electrodes are made from polyimide, a commercially available polymer, which is usually coated on silicon (Si) wafer via spin coating.^10−12^ However, it is difficult to make a uniform coating using spin coating on a cylinder electrode.^13,14^ Here, we developed pyrolyzed carbon electrodes from thin films of parylene, a biocompatible, insulating polymer, and showed they can be used with FSCV for neurotransmitter detection

Parylene, poly(*p*-xylene), is a benzene-rich polymer with chemical inertness, flexibility, and transparency and is usually used to insulate electronics.^15−17^ Parylene can be coated on different substrates via chemical vapor deposition (CVD), which guarantees uniform coating and thin deposition thickness.^18−20^ Many micro-electromechanical systems (MEMS) are insulated with parylene to form a thin film for cell co-culture and tissue barrier models.^21,22^ Parylene-C, which contains chlorine, and parylene-N, which does not, are the common forms of parylene, and other derivatives include parylene-D, VT4, and AF4.^23−25^ The Baker group previously fabricated pyrolyzed parylene-C electrodes for electrochemical detection with cyclic voltammetry (CV).^26^ However, pyrolyzed parylene has not been used with fast-scan cyclic voltammetry (FSCV) or to make electrodes for *in vivo* testing. We deposited parylene-N (PN) on etched Nb wires via CVD and used rapid thermal processing (RTP) to pyrolyze parylene into a uniformly coated thin film of carbon.

Many monoamine neurochemicals are electroactive, including dopamine (DA), serotonin, and adenosine (AD). Dopamine is a neurotransmitter that regulates movement and is involved in reward pathways.^27,28^ Serotonin is an important neurotransmitter regulating mood and depression but is difficult to detect electrochemically because it undergoes polymerization after electrooxidation, which can foul the electrode surface via π–π stacking.^29,30^ Adenosine (AD) is a neuromodulator involved in regulating vasodilation and sleep, and spontaneous transients have been measured, which are important in diseases such as ischemia.^6,31−33^ Thus, developing new electrodes for neurotransmitters involves optimizing sensitivity for different molecules and reducing fouling to enable sensitive long-term *in vivo* monitoring.

In this work, we developed a protocol to deposit PN on Nb wires and then pyrolyze it to carbon with RTP for use as an electrode to detect neurotransmitters. Surface characterization shows that defect rich carbon is produced with a high amount of oxygen functional groups. Pyrolyzed parylene-N modified electrodes (PPNMEs) were characterized for electrochemical detection of neurochemicals DA, serotonin, and AD with FSCV. Oxygen functional groups promote analyte adsorption to the electrode surface and prevent serotonin fouling. The pyrolyzed parylene-N electrodes are useful for *in vivo* detection of dopamine and adenosine, showing it is a good carbon material for general detection of neurotransmitters *in vivo*. Thus, pyrolyzed parylene is an excellent method to make a thin-film carbon electrode that is compatible with the FSCV and could be used in the future to make customized electrodes and microdevices.

## Experimental Methods

### Chemicals and Materials

Stock solutions (10 mM) were made for dopamine, serotonin, and adenosine (ThermoFisher Scientific, Waltham, WA) in perchloric acid (0.1 M). Dilute solutions for testing (1–100 μM) were made in phosphate-buffered saline (PBS) buffer pH 7.4 (131.25 mM NaCl, 3.25 mM KCl, 1.2 mM CaCl_2_, 1.25 mM NaH_2_PO_4_, and 1.2 mM MgCl_2_).

### Parylene Deposition and Pyrolysis

Niobium wires (diameter 50 μm, Advent Research Materials, Eynsham, Oxford) were etched to 600 nm in 4 M NaOH by applying a DC voltage, 2 V for 10 min. After being etched, the wires were rinsed with water and isopropanol. PN was coated on etched wires using an SCS parylene coater (PDS 2010, IN). Di-para-xylene powder, as a precursor, was vaporized in the parylene coater chamber at 150 °C under vacuum. Pyrolysis of the dimer was achieved with the application of high temperature, 650 °C, to form para-xylene, the monomer state, and those monomers then form the poly(para-xylene) structure called parylene. Parylene coating thickness can be adjusted by loading with different masses, 1, 6, and 12 g, during vapor deposition. Silicon (Si) wafers, platinum (Pt) circuit chips, and Nb wires were parylene coated with an SCS parylene coater (PDS 2010, IN). After parylene coating, Si wafers, Pt circuits, and Nb wires were treated for pre-annealing on a microhotplate at 350 °C for 10 min in an air atmosphere. To carbonize the PN coating, a rapid thermal processor (RTP) (First nano, NY) was used with these steps: (1) 600 °C in an argon atmosphere at 9 Torr for 10 min and (2) 950 °C in an argon atmosphere at 1 Torr for 10 min. RTP-PN Si wafers were used for Raman and XPS characterizations because they are flat. The conductivity test was conducted on RTP-PN Pt circuit chips. RTP-PN Nb wires were inserted into glass capillaries for insulation for a final exposed length of 50 μm for dopamine and serotonin and 100 μm for flow cell and *in vivo* adenosine, and epoxy for 5 min was used to seal the gap (J-B weld, Sulfur Springs, TX).

### Construction of CFMEs

7 μm-diameter carbon fibers (CFs) (T650-35, Cytec, Woodland Park, NJ) were inserted into glass capillaries. The capillaries were pulled in a PE-21 pipette puller (Setagaya-ku, Tokyo, Japan), and two needle-shaped microelectrodes were made. Electrodes were cut to an exposed CF length of around 50–100 μm. CFs were dipped for 30 s in a mixture of Epon Resin 828 (Danbury, CT) with 14% (w/w) *m*-phenylenediamine hardener (Acros Organics, Morris Plains, NJ) to seal the gap between the glass capillaries. Microelectrodes were rinsed in acetone for 5 s to remove excessive epoxy. CF microelectrodes (CFMEs) were left on the benchtop overnight to air-dry the epoxy at room temperature, and then, it was cured in an oven at 100 °C for 2 h and 150 °C overnight.

### Instrumentation

CVD of PN on Nb wires was performed with a parylene coater (SCS, Indianapolis, IN). Scanning electron microscopy images were collected with Merlin field emission SEM (Zeiss, Thronwood, NY) and FEI Quanta 650 SEM (ThermoFisher Scientific, Waltham, MA) with an applied accelerating voltage, 2 V, on a secondary electron detector. Analysis of graphitic features was performed by InVia Confocal Raman microscopy (Renishaw, Gloucestershire, United Kingdom). Surface characterization of elemental and functional group compositions was performed with an X-ray photoelectron spectrometer (Physical Electronics, Chanhassen, MN). Parylene thickness quantification was performed with a profilometer (KLA Tencor P-17, Milpitas, CA).

A ChemClamp potentiostat (Dagan, Minneapolis, MN) with a 1 MΩ-resistance headstage was used to collect FSCV data with a silver/silver chloride reference electrode (Pomona Electronics, Everett, WA). Silver wire was chlorinated in concentrated HCl solution with the application of 4 V. Electrochemical data was collected by applying a triangular dopamine waveform from −0.4 to 1.3 V, a scan rate of 400 V/s, and a frequency of 10 Hz. FSCV data analysis was performed on HDCV software (Department of Chemistry, University of North Carolina at Chapel Hill). Neurochemical solutions were flowed through a flow cell at 2 mL/min with the use of a six-port stainless steel HPLC loop injector with an air actuator (VICI Valco Instruments, Houston, TX). 1 M KCl was injected into glass capillaries to provide an electrical connection between the potentiostat headstage and the electrodes.

### Background-Current Subtraction

Due to the high scan rate of FSCV, 400 V/s, background currents are usually high, which makes the Faradaic current not visible. Therefore, background-current subtraction was performed while detecting neurochemicals to obtain a final current response. Because of analyte adsorption on the electrode surface, there could be a small background-subtraction error present when obtaining the final CV graphs.

### *In Vivo* Measurements

All animal experiments were performed as approved by the Animal Care and Use Committee (ACUC) of the University of Virginia. Rats (Charles River) were anesthetized with urethane (5% saline solution, 0.3 mL/100 g i.p.) before each experiment. The rectal and core body temperature was maintained at 37 °C using an isothermal pad (Delta Phase Pad; Braintree Scientific, Braintree, MA, USA). Hourly checks (paw pinch) were made of the respiration and depth of anesthesia. During surgery, a local anesthetic (bupivacaine) was used on the exposed skin and muscle tissue.

The rat was placed in a stereotaxic frame, and holes were drilled precisely in the skull to place the stimulating electrodes, working electrodes, and reference electrodes, according to the atlas of Paxinos and Watson.^34^ Specifically, the carbon-fiber working electrode was lowered into the NAc core (+1.3 mm anterior-posterior [AP], +2.0 mm medial-lateral [ML], and −7.1 mm dorsal-ventral [DV]), and the bipolar stimulating electrode (Plastics One, Roanoke, VA, USA) was lowered to the VTA (−4.7 mm AP, +0.9 mm ML, and −8.5 mm DV). The dorsoventral coordinate of the electrodes was adjusted to detect the maximum amount of stimulated dopamine release. An Ag/AgCl wire reference electrode was inserted on the contralateral side of the brain. To electrically stimulate dopamine, a constant biphasic current stimulus at +300 μA, 2 ms, and 24 pulses was delivered to the VTA by a bipolar stimulating electrode (Plastics One, Inc., Roanoke, VA, USA). Spontaneous adenosine release was measured by applying the waveform with no electrical stimulation. After each experiment, animals were euthanized using a guillotine (World Precision Instruments, Sarasota, Florida, USA).

### Brain Slice Preparation

All animal experiments were performed following the approved protocols of the Animal Care and Use Committee (ACUC) of the University of Virginia. Isoflurane was used to anesthetize a 5–8 week old wild-type C57BL/6 mouse that was decapitated quickly (Jackson Lab). aCSF was oxygenated (95% O_2_, 5% CO_2_) and held at 0–5 °C to recover the brain after being removed. The slicing stage held the brain, and a vibratome (Leica VT1000S, Bannockburn, IL, USA) was used to prepare coronal section slices (400 μm). Oxygenated aCSF (34 °C) in a water bath was utilized to equilibrate slices with caudate putamen. After transferring slices to the recording chamber, PPNMEs were inserted approximately 75 μm deep into the tissue and then equilibrated for 10–15 min. 2 mL/min was set as the flow rate to continuously perfuse oxygenated aCSF over the slice. Serotonin was stored in a glass capillary, which is close to the working electrode. A nanoliter injector (Nanoliter2020, World Precision Instrument, FL) was used to microinject a precise amount of serotonin, and CV was recorded by HDCV software.

## Results

**Figure 1:**
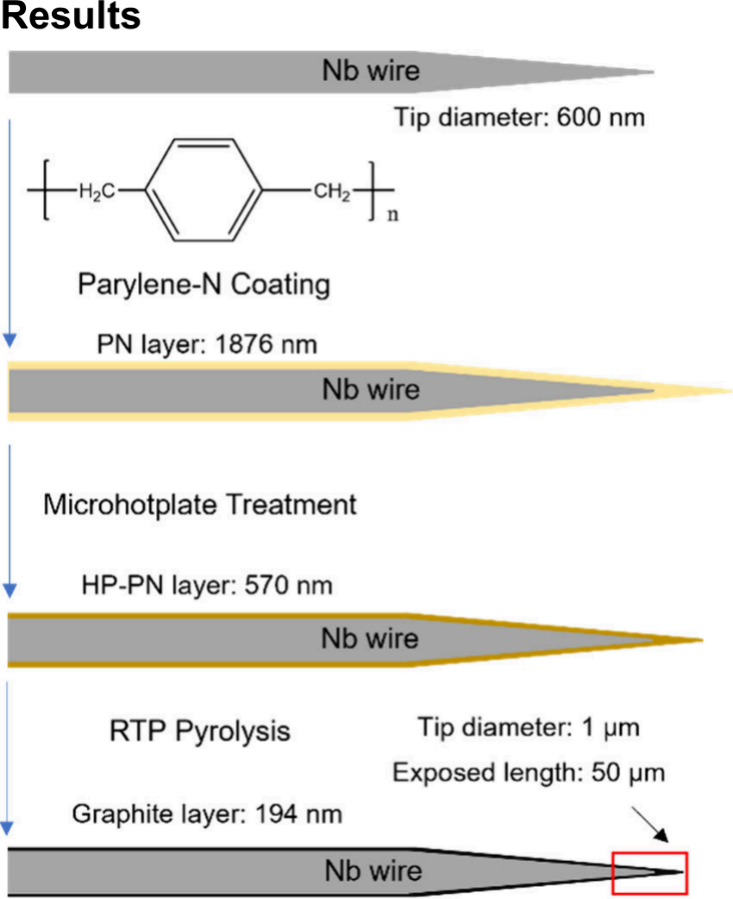
Overview of electrode fabrication. First, parylene N is deposited on a wire; then, parylene is treated on a microhotplate, and finally, pyrolysis is performed in a rapid thermal processor. The result is a thin film of graphite on a Nb wire. Thicknesses are given for 6 g of the precursor.

The overall process of electrode fabrication is shown in Figure 1. PN was deposited on the etched Niobium (Nb) wire (with a tip diameter of about 600 nm) by CVD, and the coating thickness is about 1876 nm for 6 g of precursor. The PN-coated Nb wire was then heated on a microhotplate at 350 °C (HP-PN), which shrinks the coating to 570 nm. Pyrolysis was performed using RTP at 600 °C for 10 min and then 950 °C for 10 min, parameters that were optimized for pyrolysis of 3D printed electrodes made of photoresist.^35^ Future studies could examine the effect of the pyrolysis parameters on the electrochemical performance. The coating after RTP is conductive graphite, and the final thickness is about 194 nm. Thus, a thin-film carbon electrode is achieved.

We tested using different masses of parylene precursor, 1, 6, and 12 g, to understand the coating thickness and conductivity at various steps. The deposited parylene thickness on flat silicon wafers was consistent and ranged from 327 nm to 3.4 μm (Table 1) and is linear with the precursor mass (Figure S1). Thicknesses should be similar on Nb wires, but they are difficult to measure on a wire. After heating on the microhotplate, the thickness of PN shrunk due to weight loss and varied from 122 to 867 nm. After the pyrolysis of parylene with RTP, the thickness decreased even more and varied from 81 to 476 nm. Thus, this method leads to nanoscale films of carbon on the surface.

**Table 1:** Parylene Coating Thickness with Various Masses at Different Stages of Treatment^a^

Precursor Mass (g)	Deposited Thickness (nm)	Thickness after Hotplate (nm)	Thickness after Pyrolysis (nm)
1	327 ± 21	122 ± 16	81 ± 10
6	1876 ± 96	570 ± 43	194 ± 20
12	3408 ± 158	867 ± 73	476 ± 35

a Error is SEM, *n* = 3.

**Figure 2:**
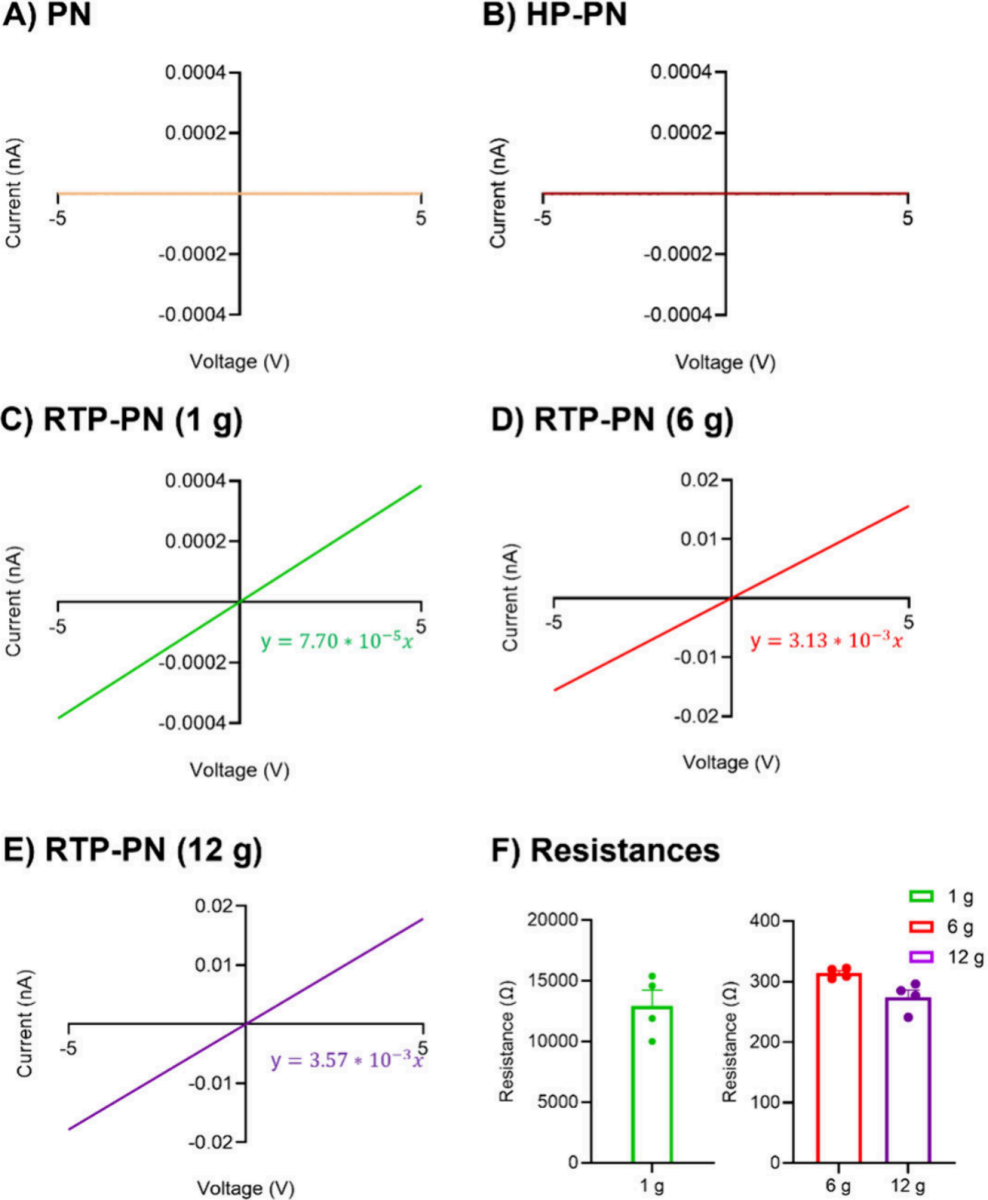
Electrical conductivity tests of (A) PN (yellow line); (B) HP-PN (brown line); (C) RTP-PN (1 g); (D) RTP-PN (6 g); and (E) RTP-PN (12 g) on Pt electric circuits. (F) Resistances of RTP-PN (1, 6, and 12 g) (*n* = 4; Error bars are SEM).

PN was deposited on a printed platinum (Pt) circuit with gaps between the electrodes, and conductivity was measured by applying a voltage and measuring current. The slope was used to determine the electrical resistances of different polymers. PN is an insulating polymer that has no electrical conductivity because of the high resistance, and the plot in Figure 2A confirms this. There is also no conductivity for HP-PN (Figure 2B), so the polymer was not yet graphitized after the hotplate treatment. The RTP-PN samples were conductive because the plots have a slope that corresponds to the resistance (slope = 1/*R*, Figure 2C–E). Thus, it takes a high temperature to carbonize parylene and make it conductive. Conductivity at RTP-PN samples was measured multiple times (Figure S2), and their resistances were calculated and shown in Figure 2F. We simulated the circuit with an RTP-PN (6 g) thickness, 194 nm, and resistance at 320 Ω on COMSOL software. The estimated conductivity is about 3000 S/m, which is within the conductivity range of glassy carbon wires, 477–18100 S/m.^36^ Generated glassy carbon when annealed at 850 °C for 4 h possesses a conductivity of 3200 S/m, which is similar to that of RTP-PN (6 g).^37^

PN underwent volumetric shrinkage, which is about 89.7% for PN-coated Nb wires (6 g), and structural reformation into graphite after high temperature annealing.^26,38,39^ The mechanism is likely high temperature breaking of carbon bonds and reaction of benzene rings to form graphite.^40^ Tai’s group saw similar changes in parylene-C (PC) coating thickness and resistivity when different temperatures were applied, 0 °C to 900 °C.^38^ PC’s resistivity dramatically decreased with raising temperature, especially above 500 °C, similar to what we observed in the conductivity test where conductivity increased after pyrolysis with RTP-annealing steps at 600 and 950 °C. All RTP-PN with different amounts of precursor have similar conductivities, so we selected RTP-PN (6 g) for further investigation. FSCV waveforms with switching potentials over 1.3 V are known to break down carbon material, so a slightly thicker film might be more stable over time (Figure S3).^41^

**Figure 3:**
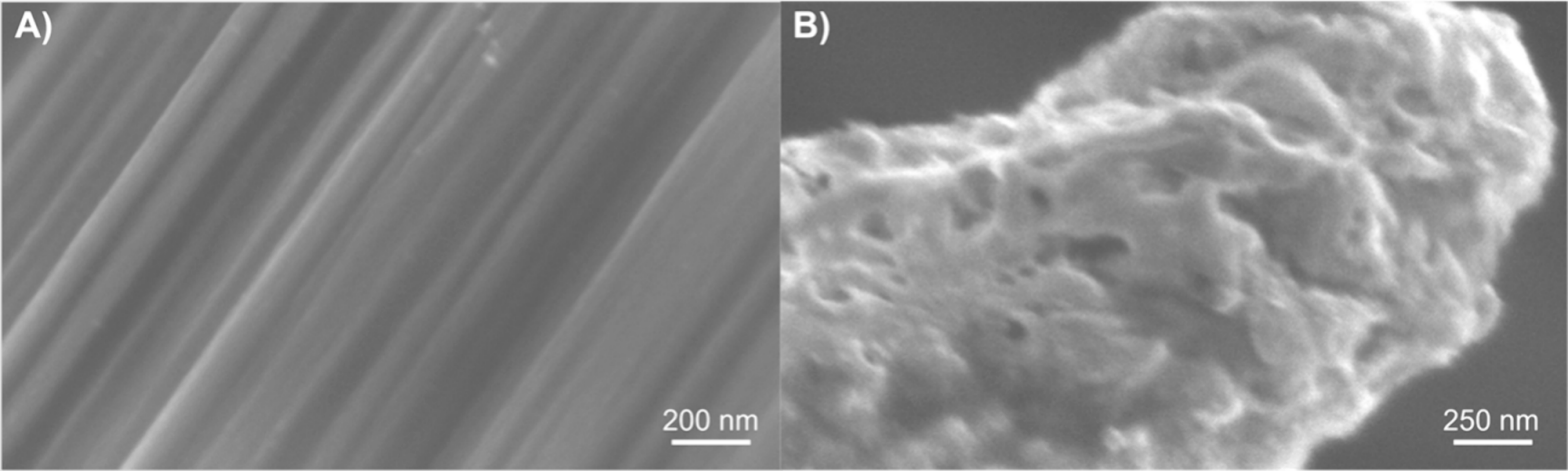
SEM images of the (A) surface of CF and the (B) surface of RTP-PN (6 g).

We characterized the surfaces by SEM (Figure 3A,B, respectively). The CF structure is mainly smooth, with some grooves. CFs are about 7 μm, and the overall diameter of the PPNME on the etched Nb wire is about 1 μm (6 g deposited), which is smaller than that of CF. Therefore, the PPNME could be localized better in specific brain regions and cause less inflammation in tissue. The nanopores of the PPNME are larger and deeper than the grooves on the CFME, which help restrict diffusion of neurochemicals that enter the nanopores.^42^ However, the pores are smaller than those on carbon nanotube yarn microelectrodes (CNTYMEs), and thus, the effects of thin layer diffusion in nanopores at the PPNMEs are expected to be more limited compared to carbon nanotube yarn microelectrodes (CNTYMEs).^42^

**Figure 4:**
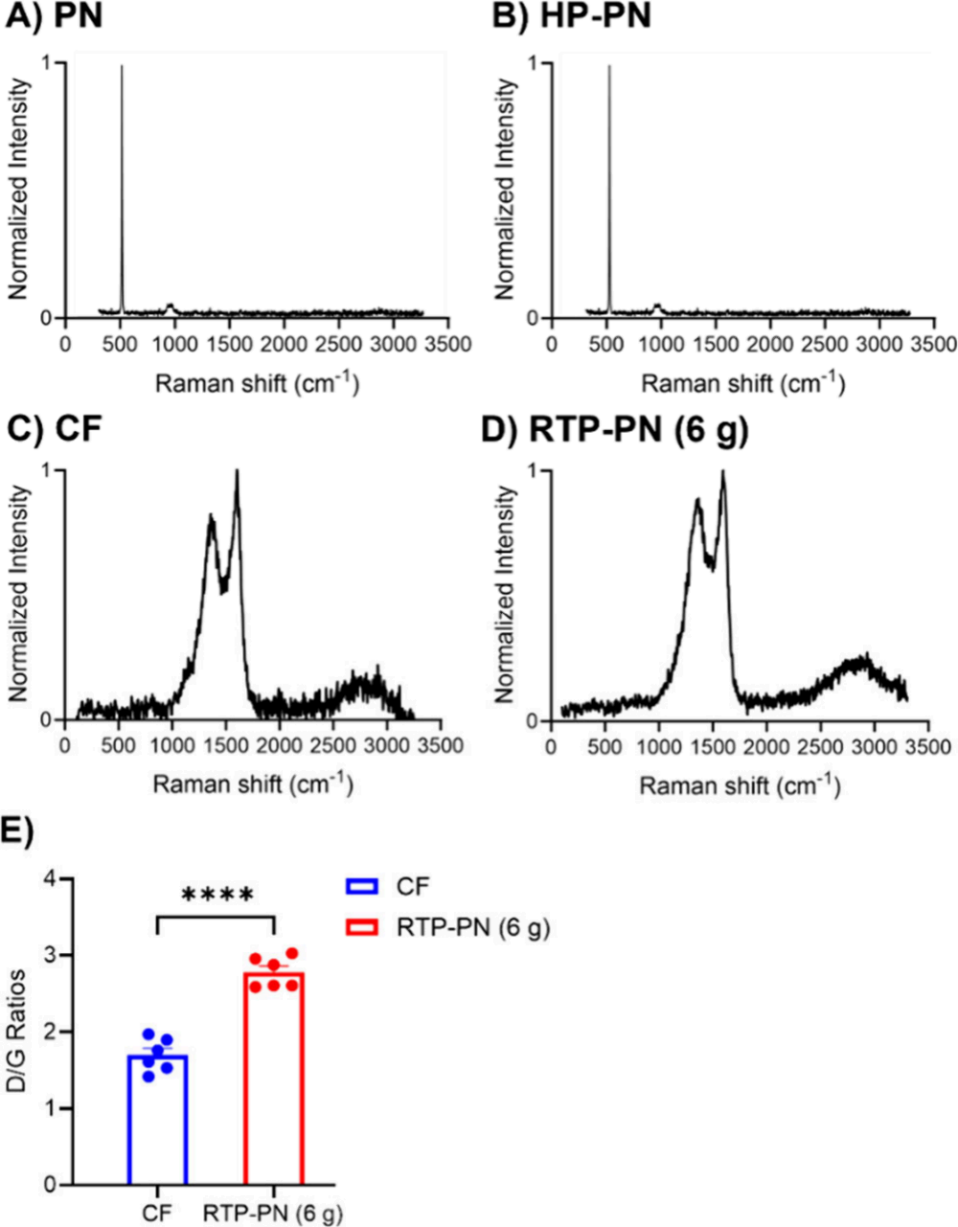
Raman spectra of (A) PN, (B) HP-PN, (C) CF, and (D) RTP-PN (6 g) on Si wafer. The D, G, and 2D peaks are located at 1360 cm^–1^, 1580 cm^–1^, and 2860 cm^–1^, respectively. (E) Bar graphs of D/G ratios of CF and RTP-PN (6 g) (*n* = 6, *t* test, ****p < 0.0001; Error bars are SEM).

Raman spectra were analyzed to study the graphitic features of all materials. The defect sites at the boundaries, such as sp^3^ carbon, oxygen functional groups, or doping, generate a D band (∼1360 cm^–1^). sp^2^ graphitic carbons generate a G band (∼1580 cm^–1^), which is formed from basal planes.^43−45^ The 2D peak (2860 cm^–1^) helps identify the material as graphene.^46−48^Figures 4A and 3B show PN before and after heating at 350 °C on the microhotplate for 10 min. Heating on the hotplate does not cause graphitization, as there is no obvious difference between Figure 4A and B, which have only Si peaks from the wafer (522 cm^–1^ and 961 cm^–1^), originated from the Si wafer.^49^ There are no graphitic features on either Raman spectrum, which indicates that PN, a polymer, possesses no graphitic structure before RTP. RTP treatment induces full carbonization of PN, with the presence of D, G, and 2D peaks. The high temperature provides energy to change the polymer structure into multilayer graphene, which is confirmed by the presence of a 2D peak. The disorder level of carbon materials was determined by calculating the ratios of the D peak and G peak areas, and they were 1.9 for CF and 2.6 for RTP-PN. A higher D/G ratio means that there are more defect sites on RTP-PN, and a decreased number of basal planes might decrease pi stacking with molecules, which could improve anti-fouling behavior. Average D/G ratios of CF and RTP-PN (6 g) are plotted in a bar graph, Figure 4E.

**Figure 5:**
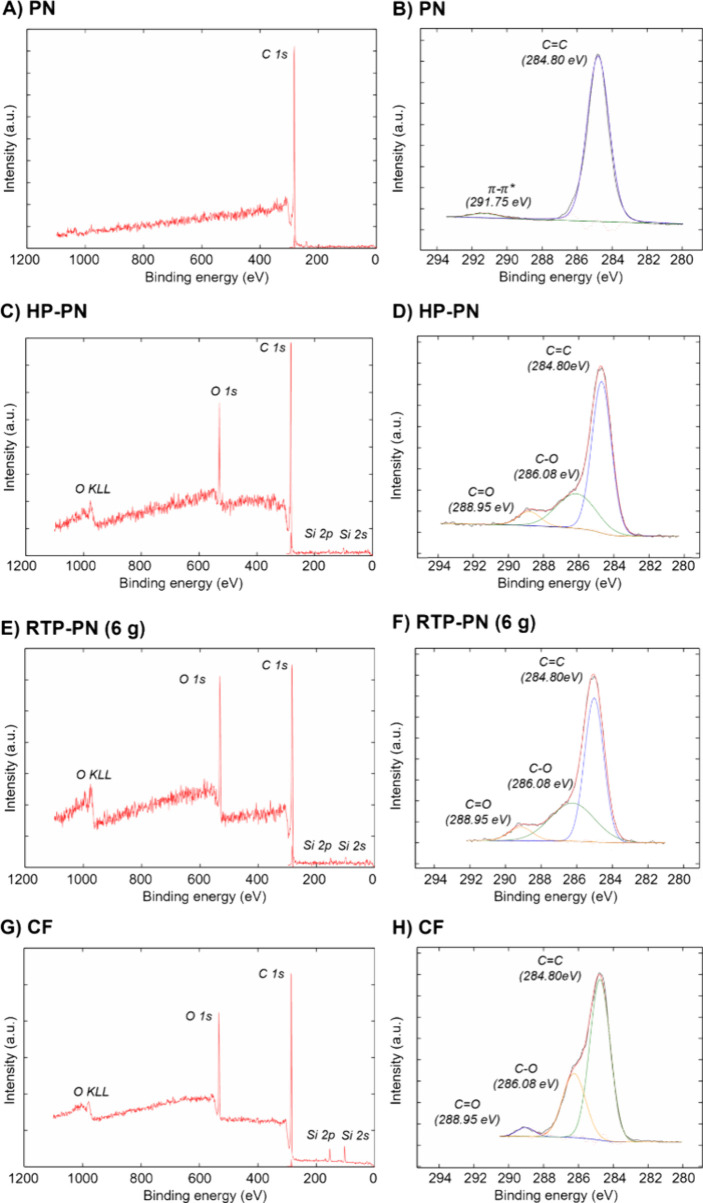
XPS analysis of elemental compositions (A) PN, (C) HP-PN, (E) RTP-PN (6 g), and (G) CF and functional groups (B) PN, (D) HP-PN, (F) RTP-PN (6 g), and (H) CF on Si wafers.

Raman spectroscopy shows that RTP-PN is a defect-rich graphitic material. XPS was utilized to characterize the specific surface elemental compositions and functional groups of the pyrolyzed PN. PN is a benzene-rich polymer with no oxygen functional groups, and π–π stacking originates from the polymer overlapping (Figure 5A,B). After pre-annealing PN with a microhotplate in an air atmosphere, sp^2^ carbon bonds were broken and C–O and C=O bonds were formed; oxygen was introduced into the PN polymer structure (Figure 5C,D). However, the insertion of oxygen atoms in PN does not induce conductivity, as no graphitic carbon was formed during microhotplate treatment. π–π stacking is absent after pre-annealing as the peak at 292 eV is not present. After RTP-annealing, all carbon–oxygen functional groups are still present (Figure 5E,F). Scan rate tests confirm adsorption-controlled behavior, and surface oxygen groups can contribute to adsorption of dopamine and other neurochemicals on carbon electrodes (Figure S4).^8,10^

Table 2 shows the atomic percentages of carbon and oxygen on the surfaces of PN, HP-PN, RTP-PN (6 g), and CF. There was no oxygen present on the PN surface, which only contains carbon. After pre-annealing on the hotplate, oxygen was involved in PN. After pyrolysis with RTP, carbon bonds were broken and surface atomic percentage dropped correspondingly. Compared with RTP-PN (6 g), CF possesses less oxygen content.

**Table 2:** Functional Group Percentages of PN, HP-PN, RTP-PN, and CF

Functional Group	Binding Energy (eV)	PN (Area %)	HP-PN (Area %)	RTP-PN (6 g) (Area %)	CF (Area %)
C=C	284.80	96.7	65.1	57.7	66.9
C–O	286.08	0	28.8	35.3	29.9
C=O	288.95	0	6.0	7.1	3.2
π–π*	291.80	3.3	0	0	0

Table 3 displays the area percentages of each functional group on XPS spectra in Figure 5C,E,G by curve fitting. Carboxyl groups promote neurochemical adsorption.^4^ PN is a benzene-rich polymer with C=C present in the polymer structure.^50,51^ Because of the overlapping of the polymer layers, π–π stacking is shown in the XPS spectra. However, after HP pre-annealing, the stacking structure on PN surface was broken and oxygen-containing functional groups were formed, which resulted in the disappearance of π–π stacking.^49,50^ After application of high temperature with RTP, the π–π stacking decreased, while the oxygen-containing groups increased. The XPS analysis, coupled with Raman spectra, shows that the pyrolyzed parylene has increased functional group formation during HP pre-annealing and RTP annealing. Oxygen content and functional groups of RTP-PN (6 g) are more than those of CF, which should benefit electrochemical detection of more neurotransmitters.

**Table 3:** Functional Group Analysis of PN, HP-PN, RTP-PN, and CF

Element	PN (Atomic %)	HP-PN (Atomic %)	RTP-PN (6 g) (Atomic %)	CF (Atomic %)
C	100	85.8	79.1	86.6
O	0	14.2	20.9	13.4

**Figure 6:**
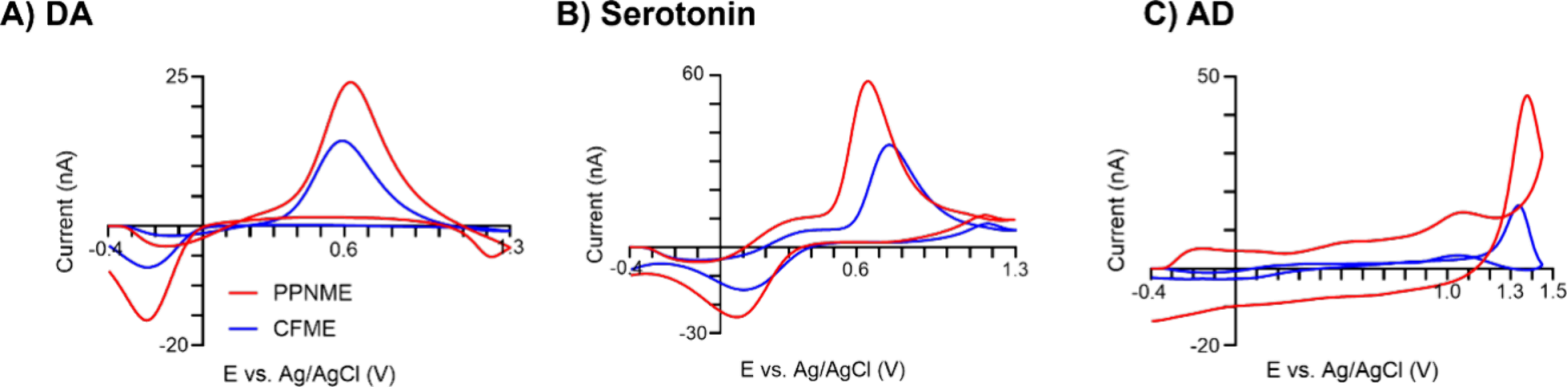
Electrochemical detection (CVs) of dopamine (DA), serotonin, and adenosine (AD) at CFMEs (blue) and PPNMEs (6 g) (red) for (A) 1 μM DA, (B) 1 μM serotonin, and (C) 1 μM AD.

CFMEs and PPNMEs were used to test the electrochemical detection of various neurotransmitters including 1 μM DA, serotonin, and AD (Figure 6). The magnitudes of neurochemical oxidation currents on PPNMEs are higher than those on CFMEs. The PPNMEs have enhanced oxygen functional groups, which increase the electrostatic force between the positively-charged analytes and the surface, and thus, there is good sensitivity.^4,6,52^ The shapes of the CVs for dopamine and serotonin on PPNMEs were similar to those of CFMEs. The primary peak for adenosine is located on the back scan due to the high scan rate in FSCV.^53^ The secondary peaks for AD oxidation currents were further enhanced from 20% of the primary current at CFMEs to 33% at PPNMEs.

**Figure 7:**
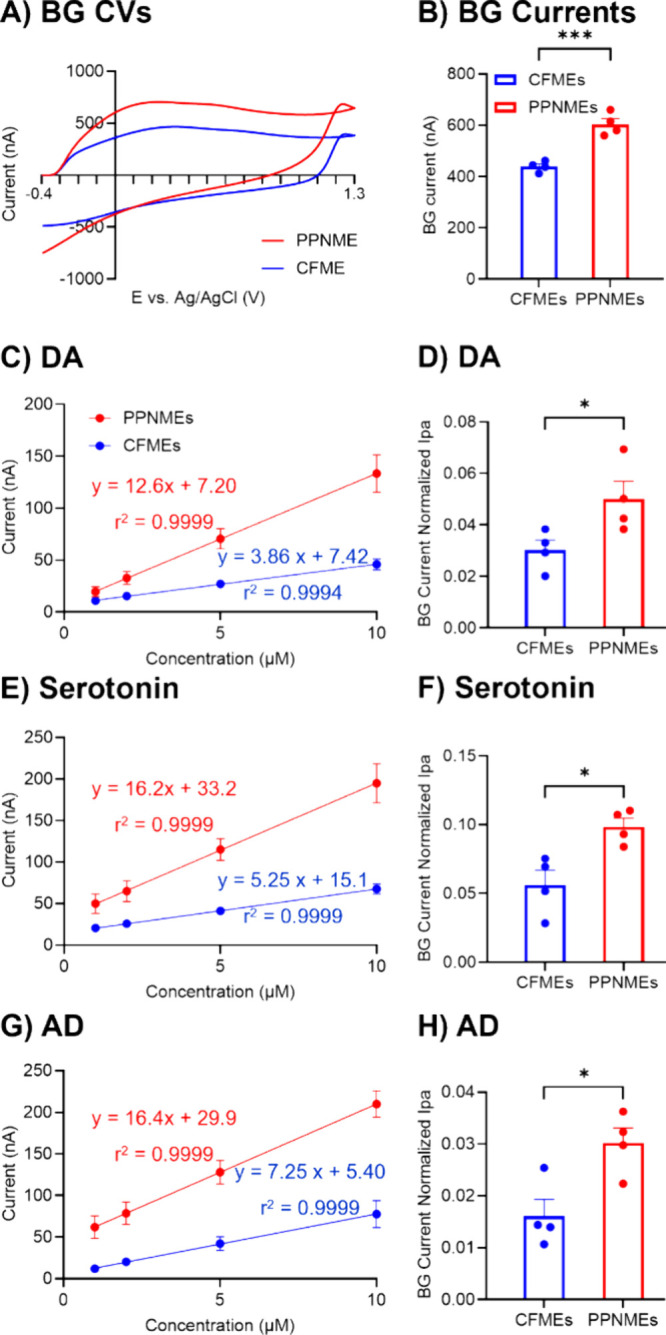
Background currents of electrodes and background-normalized currents and sensitivity tests of DA, serotonin, and AD on CFMEs and PPNMEs. (A) Background CVs of CFME and PPNME. (B) Bar graphs comparing background currents. (C) Sensitivity for 1–10 μM DA. (D) Background normalized current comparison for 1 μM DA. (E) Sensitivity for 1–10 μM serotonin. (F) Background normalized current comparison for 1 μM serotonin. (G) Sensitivity for 1–10 μM AD. (G) Background normalized AD currents for 1 μM adenosine (all bar graphs: *n* = 4, *t* tests, *p < 0.1, ***p < 0.001; Error bars are SEM).

Figure 7A shows the CVs of CFME and PPNME background currents. In Figure 7B, the PPNME surface area is generally about 1.4 times higher than the CF background signal, which shows a larger surface area for neurochemical detection. In Figure 7C,E,G, Faradaic currents for each analyte were plotted against concentration and the slope in the linear range was used to quantify sensitivity (higher concentrations shown in Figure S5). DA, serotonin, and AD are analytes that are positively charged at physiological pH, 7.4, and can be adsorbed to the electrode surface with the applied potential, −0.4 V. Generally, PPNMEs have higher slopes and higher sensitivities than CFMEs, about 2–3 times, which is larger than the difference in background currents. As PPNMEs have larger surface areas, Faradaic currents were divided by background currents to normalize the surface area. PPNMEs are compared to CFMEs in Figure 7D,F,H. Even correcting for background current, which is proportional to surface area, the signal for all 3 neurochemicals is higher for PPNMEs.

**Figure 8:**
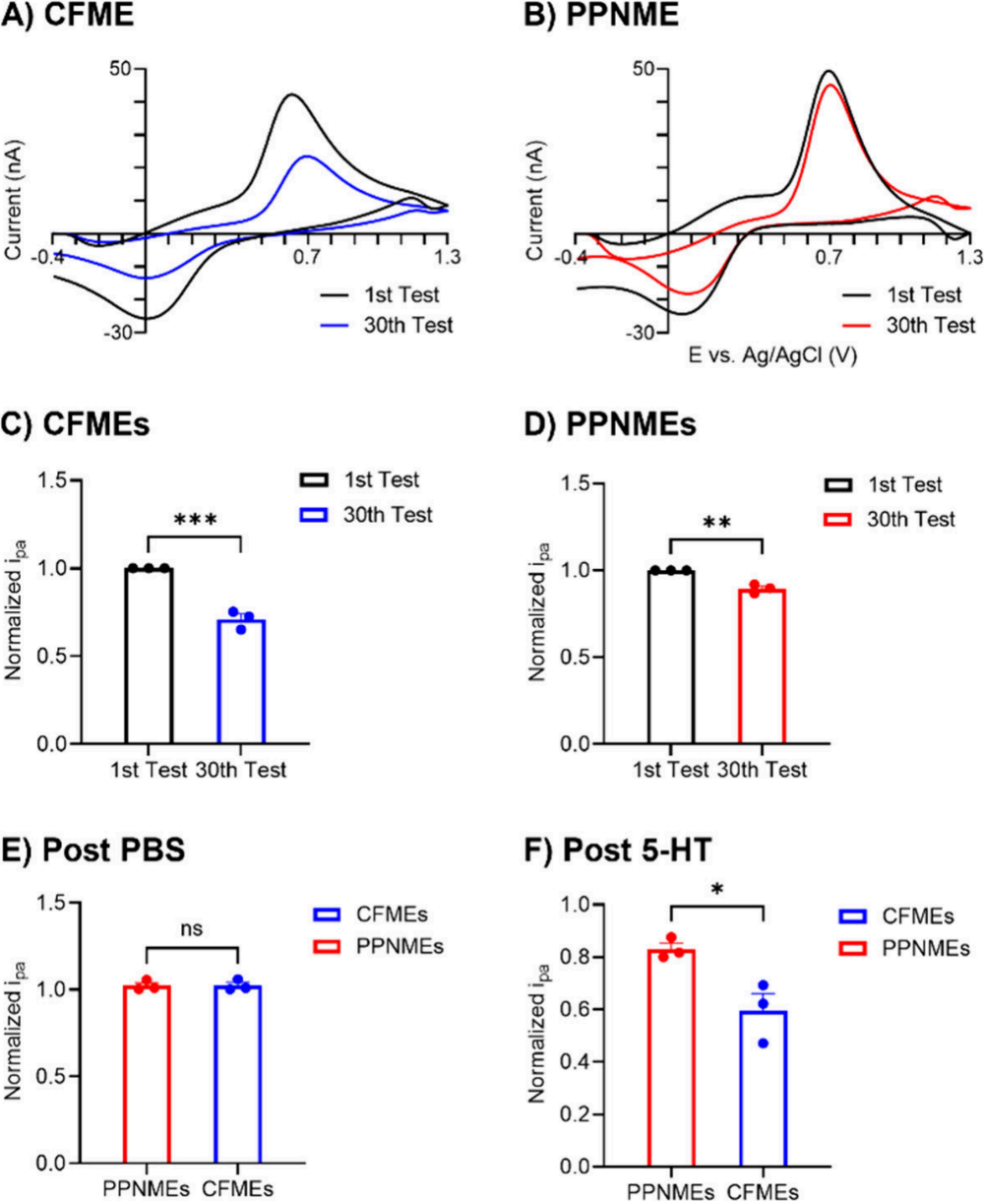
Serotonin fouling test on CFMEs and PPNMEs. (A) CVs of the 1^st^ and 30^th^ injections of 1 μM serotonin on CFMEs. (B) CVs of the 1^st^ and 30^th^ injections of 1 μM serotonin on PPNMEs. (C) Bar graph 1^st^ and 30^th^ injections of 1 μM normalized serotonin currents on CFMEs. (D) Bar graph 1^st^ and 30^th^ injections of 1 μM normalized serotonin currents on PPNMEs. (E) Bar graph of 1 μM normalized serotonin oxidation signals on CFMEs and PPNMEs after soaking in PBS buffer for 1 h. (F) Bar graph of 1 μM normalized serotonin oxidation signals on CFMEs and PPNMEs after soaking in 1 μM serotonin for 1 h (*n* = 3, *t* test, *p < 0.1, **p < 0.01; Error bars are SEM).

Fouling is a serious problem during electrochemical detection of some molecules, particularly serotonin.^54,55^ Serotonin molecules are polymerized with the application of the dopamine waveform, and the polymer attachment on the electrode surface blocks the active sites for neurochemical adsorption, which will decrease detection sensitivity.^29,30,54^ With a rich amount of defect sites, we hypothesize that less fouling should be present on PPNMEs because of the decreased basal planes, which will allow less π–π stacking between the serotonin polymer and the electrode surface. 1 μM serotonin was injected in both electrodes 30 times every 15 s, and serotonin currents at CFMEs and PPNMEs dropped about 50% and 5%, respectively (Figure 8).

To further test whether PPNMEs could resist serotonin fouling, longer-term fouling experiments were performed on both electrodes. As a control, electrodes were tested with serotonin before and after the waveform was applied in PBS buffer for 1 h, and the current does not change (Figure 8E). However, after applying the dopamine waveform for 1 h in 1 μM serotonin, the oxidation currents for CFMEs dropped significantly, about 50%. In contrast, at PPNMEs, the current dropped only about 20%, meaning 80% sensitivity was retained (Figure 8F). The high density of nanopores and rich defect sites on the PPNME surface will promote anti-fouling properties because there would be reduce π–π stacking between electrode surface and serotonin polymer.^4,6,30,56^

**Figure 9:**
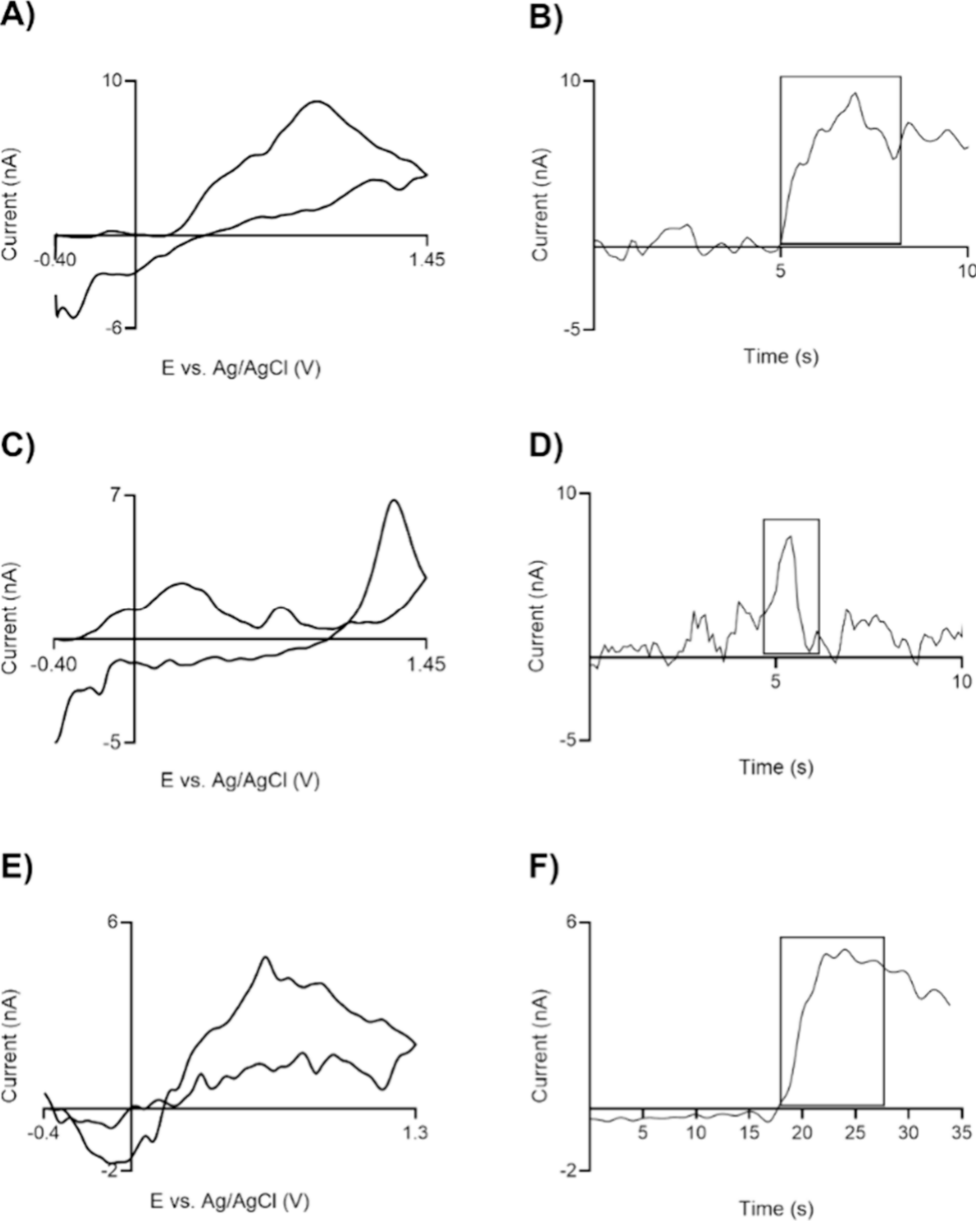
*In vivo* dopamine, adenosine, and serotonin testing with PPNMEs. (A) Stimulated DA CV; (B) DA IT curve. (C) Transient (unstimulated) AD CV; (D) AD IT curve. (E) Applied serotonin CV; (F) serotonin IT curve.

PPNMEs were employed *in vivo* to demonstrate their efficacy in detecting neurochemicals. For dopamine testing, stimulated dopamine release was evaluated in the mesolimbic circuit by applying electrical stimulation to the ventral tegmental area (VTA) and measuring dopamine in the nucleus accumbens (NAc) of rats. Figure 9A illustrates the cyclic voltammogram of dopamine released at about 800 nM upon electrical stimulation. As shown in the *i* vs *t* curve in Figure 9B, the peak current increases with electrical stimulation, indicating dopamine release. Peaks in the CV are a bit wider *in vivo*, likely due to biofouling or possible background subtraction errors due to ionic changes that occur, changing the background current. As the testing was performed in the short-term, about 2 h, no dramatic fouling happens on the reference electrode, Ag/AgCl (Figure S6), which would disturb the neurotransmitter detection. Therefore, no Nafion coating needs to be applied to the reference electrode.^57,58^

Additionally, we continuously collected data and observed spontaneous adenosine transients with the PPNMEs.^33,59,60^ A representative transient, about 858 nM, is depicted in Figure 9C (cyclic voltammogram showing adenosine-dopamine corelease) and Figure 9D (*I* versus *t* curve showing primary oxidation peak). These transients occurred without electrical stimulation and lasted approximately 1–2 s. As indicated by the cyclic voltammograms, the PPNMEs successfully measured both dopamine and adenosine, demonstrating their utility for detecting neurochemicals *in vivo*. As the tested region that releases serotonin is too small to perform electrical stimulation, we tested PPNMEs in the mouse brain slice (caudate putamen). PPNMEs were inserted into the tissue. A nanoliter injector was used to microinject serotonin close to the working electrode. Figure 9E,F presents exogenously applied serotonin CV and the *I* verses *t* curve, and 832 nM serotonin was detected. While much of these data are similar to detection at CFMEs, the future possibility is to use PPN to make different geometries, and so, this PPN will prove useful for *in vivo* measurements. For example, since PN is an insulator, if part of it can be masked and then pyrolyzed, perhaps using a laser or focused-ion beam milling, one could fabricate arrays and other geometries that are not possible to fabricate with carbon fibers.

## Conclusions

RTP treatment of parylene N provides a method to fabricate microelectrodes with a thin film of carbon to sensitively track neurochemicals. Enhanced defect sites increased the detection sensitivity for dopamine, serotonin, and adenosine. PPNMEs possess an antifouling property, which makes modified electrodes suitable for sensitive and long-term *in vivo* testing. Nb wires deposited with PN were etched to the nanoscale, which makes them an attractive option for tissue applications. These electrodes can detect *in vivo* dopamine and adenosine release and thus are suitable for monitoring real-time release of neurotransmitters in tissue. Future work can examine other uses of this thin film electrode technology to customize different electrodes and microdevices.

## Supporting Information Available

Figure S1: Thicknesses of PN and PPN plotted against PN precursor weight; Figure S2: PN, HP-PN, and RTP-PN samples on the Pt circuit; Figure S3: Stability test of PPNMEs; Figure S4: Scan-rate testing (50–1000 V/s) on CFMEs and PPNMEs; Figure S5: Concentration tests of DA, serotonin, and AD; Figure S6: CVs of normalized DA currents before and after Ag/AgCl wire insertion into the brain slice (PDF)
