# Supplementary material for: Pyrolyzed Parylene-N for in Vivo Electrochemical Detection of Neurotransmitters
Source: ACS Electrochem. 2025 Mar 27;1(5):730–40. doi: 10.1021/acselectrochem.4c00180 (PMC12051191; doi:10.1021/acselectrochem.4c00180)
Supplement: Supplementary file 1 — ec4c00180_si_001.pdf [file ec4c00180_si_001.pdf]

Supporting Information

Pyrolyzed Parylene-N for *In Vivo* Electrochemical Detection of Neurotransmitters

He Zhao [a], Owen Markow [a], Greatness Olaitan [a], Eric D. Donarski [a], Kevin C. Lester [b], Nickolay V. Lavrik [b], B. Jill Venton [a,*]

[a] Department of Chemistry, University of Virginia, Charlottesville, Virginia, 22904, USA

[*]Corresponding author

E-mail: bjv2n@virginia.edu

[b] Center for Nanophase Materials Sciences, Oak Ridge National Lab, Tennessee, 37831, USA

Table of Contents

Thicknesses of PN and PPN plotted against PN precursor weight S2

PN, HP-PN, and RTP-PN coatings on Pt circuits..S3

Stability test of PPNMEs S4

Scan-rate testing (50-1000 V/s) S5

Concentration tests S6

CVs of normalized DA currents before and after Ag/AgCl wire insertion into the brain slice S7

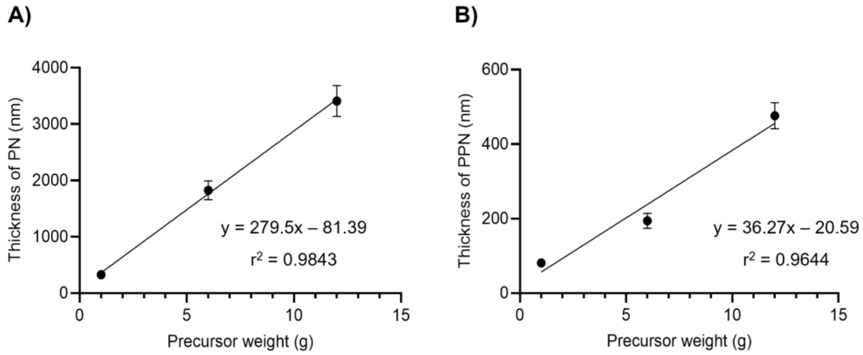

Figure S1. Thicknesses of PN and PPN plotted against PN precursor weight.

Fig. S1 plotted thicknesses of (A) PN and (B) pyrolyzed PN against PN amount (1 g, 6 g, and 12 g). (A) The deposited polymer is linear with precursor weight for the tested amounts. (B) After pyrolysis, PN undergoes volumetric shrinkage and thicknesses of pyrolyzed parylene-N are still linear, but there is slightly more variance and a lower R^2 value of 0.9644.

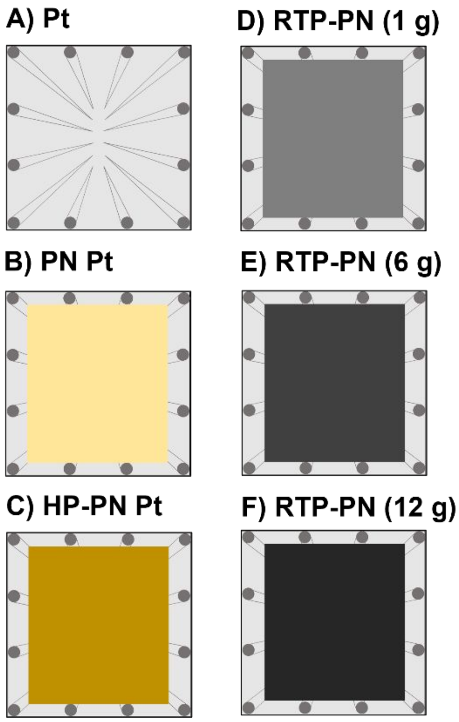

Figure. S2. (A) Pt circuits (B) Pt circuits with PN (C) Pt circuits with HP-PN (D) Pt circuits with RTP-PN (1 g) (E) Pt circuits with RTP-PN (6 g) (F) Pt circuits with RTP-PN (12 g)

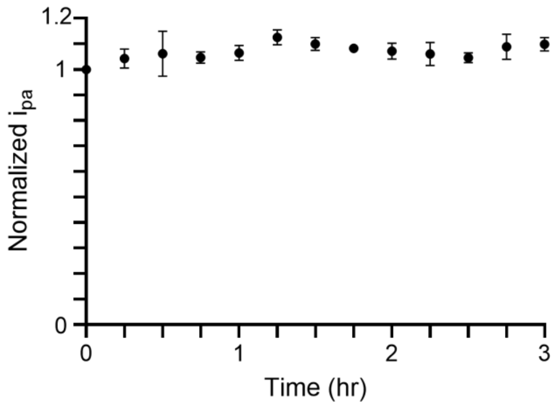

Figure S3. Stability test of PPNMEs.

To ensure RTP-PN can be used for long-term electrochemical detection, PPNMEs were applied in the flow cells for testing DA for 3 hr. Normalized currents responses were recorded for each 15 min and plotted against the time in Figure. S4. After 3 hr, Faradaic currents at 3 hr remained similar to the original currents. Therefore, long-term electrochemical detections can be performed on PPNMEs.

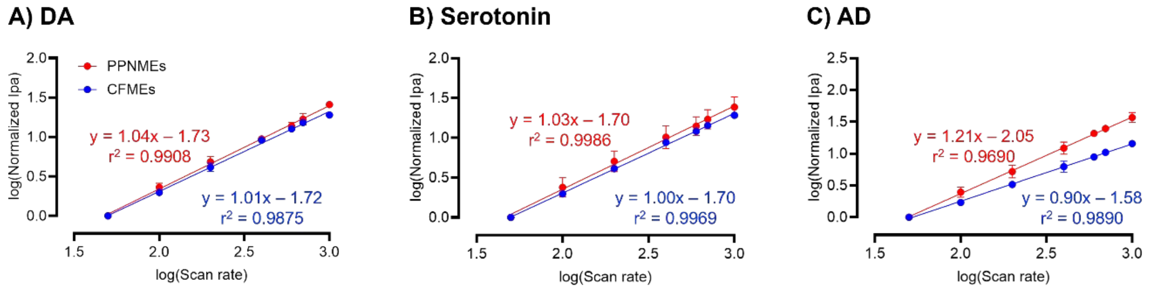

Figure S4. Scan-rate testing (50-1000 V/s) of (A) DA (B) serotonin (C) AD on CFMEs and PPNMEs.

Different scan rates, ranging from 50 to 1000 V/s, were applied to detect neurochemicals. Log normalized oxidation currents for each analyte were plotted against log scan rate to determine the slope. If the slope is 0.5, the detection is diffusion controlled; if it is 1.0 then detection is adsorption-controlled.[4,55] The slopes were about 1 for monoamines DA and serotonin. The slopes on PPNMEs indicate that RTP-PN induced adsorption-controlled detection for DA, serotonin, and AD. AD adsorption was promoted on PPNMEs compared to the electrochemical performance on CFMEs, which induced diffusion-and-adsorption-mixed controlled detection.

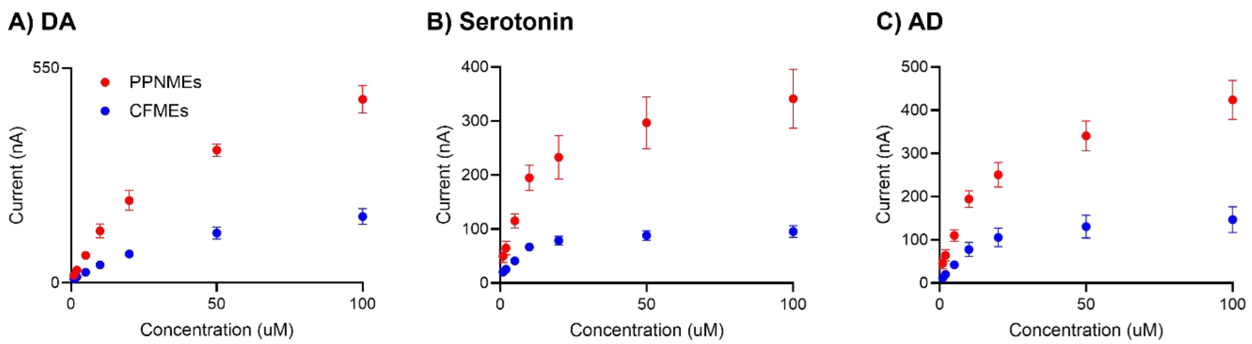

Figure S5. Concentration tests of DA, serotonin, and AD. (A) 1-100 μM DA (B) 1-100 μM serotonin (C) 10-500 μM AD.

CFMEs and PPNMEs were used to test the wide concentration range of DA, serotonin, and AD (Fig. S4). Higher oxidation currents were detected at PPNMEs. Oxidation signals reached a plateau when analyte concentrations are over 10 μM. As active sites on the electrode surface are limited, once all the sites on the surface are saturated, then kinetics are limited by diffusion to the surface instead. Therefore, the linear range was 1-10 μM for all neurochemicals.

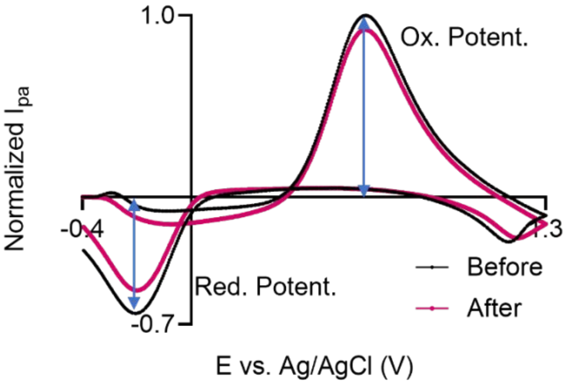

Figure S6. CVs of normalized DA currents before and after Ag/AgCl wire insertion into the brain slice.

To test if biofouling, during *in vivo* or puff-on experiments, affects the reference electrode and electrochemical detection, an Ag/AgCl wire was inserted into the brain slice for 2 hr. Normalized DA signals were recorded before and after Ag/AgCl wire insertion into the tissue and plotted in Fig. S6. No obvious change on current, oxidation potential, or reduction potential is observed in CV graphs, which indicates that electrodes in short-term *in vivo* testing are not severely affected by biofouling.
